# Mnemonic Discrimination Performance in Anxiety and Depression: A Systematic Review

**DOI:** 10.1155/da/8826935

**Published:** 2026-02-04

**Authors:** Alexandra Kaszás, Szabolcs Kéri

**Affiliations:** ^1^ Department of Cognitive Sciences, Budapest University of Technology and Economics, Műegyetem rakpart 3, Budapest, 1111, Hungary, bme.hu; ^2^ University of Tokaj, Eötvös út 7, Sárospatak, 3950, Hungary; ^3^ Department of Physiology, Albert Szent-Györgyi Medical School, University of Szeged, Szeged, 6720, Hungary, u-szeged.hu

**Keywords:** anxiety, depression, mnemonic discrimination, pattern separation, systematic review

## Abstract

Anxiety disorders and depression are the most frequently diagnosed mental illnesses and are highly comorbid. Both have been linked to memory impairments, albeit the relationship between them remains unclear and understudied. Our review aims to investigate behavioral pattern separation performance in individuals with varying levels of anxiety and depression. We included studies where mnemonic discrimination performance is measured using mnemonic discrimination tasks designed to directly measure behavioral pattern separation, while symptoms of anxiety and/or depression are assessed using any validated and recognized scales or inventories. We only included quantitative studies. Fixed scientific databases and artificial intelligence were systematically searched, identifying nine studies on anxiety and 14 on depression. Anxiety studies presented conflicting evidence, but a trend showed optimal mnemonic discrimination with threat‐based encoding and safe retrieval. Conversely, depression studies consistently suggested a negative relationship between symptom severity and pattern separation performance. Additionally, pattern separation appeared enhanced for negative stimuli and impaired for neutral stimuli in individuals with higher levels of depression. However, no clear differences were observed between clinically diagnosed groups and healthy controls. Methodological inconsistencies in anxiety studies present challenges for interpretation. Similarly, the effects of medication and the heterogeneity of clinical groups pose limitations to depression studies. Trends were identified, but further investigation with unified methodologies and clinical groups is needed to understand the relationship between anxiety, depression, and mnemonic discrimination performance.

## 1. Introduction

Anxiety and depression are among the most prevalent mental health disorders worldwide, each profoundly affecting emotional, cognitive, and behavioral functioning [1, 2]. Although distinct in symptomatology and neural substrates, these disorders often co‐occur and share overlapping features, particularly in memory processing disruptions. Understanding their relationship with specific memory mechanisms, such as pattern separation, may shed light on the cognitive deficits observed in these conditions.

### 1.1. Anxiety

Anxiety is a response evoked by a diffused, unpredictable threat, resulting in affective, cognitive, and physiological changes. Fear, which is a response to a certain predictable threat, is distinct from anxiety at neural, pharmacological, and behavioral levels [3–5]. While anxiety bears evolutionary importance and can be adaptive in uncertain and unfamiliar situations to help us detect and avoid danger [6, 7], it reaches a diagnostic threshold when the given response becomes persistent and excessive and leads to an impairment in functioning, including social and occupational aspects. Anxiety disorders are distinguished by disproportionate and enduring fear‐related reactions and behaviors that surpass what is reasonably suitable for the given context or situation [1]. In 2019, over 300 million people suffered from an anxiety disorder worldwide, which makes them the most common mental disorders [2].

### 1.2. Depression

Depression is a prevalent and significant medical condition that has adverse impacts on one’s emotions, cognitive processes, and behavior. It induces feelings of sadness and a diminished interest in previously enjoyable activities. This condition can give rise to a range of emotional and physical challenges, impairing one’s capacity to perform effectively both in professional and domestic settings. Statistics indicate that approximately one in 15 adults (6.7%) grapples with depression in any given year, and one in six individuals (16.6%) will encounter depression at some point in their lifetime [1].

### 1.3. Memory Disruptions in Anxiety

Memory [8] could be defined as the ability to encode, store, and retrieve information or representations of our experiences. While the facilitation of threat detection in uncertain and unpredictable situations is evolutionary adaptive, the changes caused by both induced [9, 10] and pathological anxiety [11, 12] can lead to deficits in memory functioning. Anxiety, particularly when characterized by heightened state anxiety, is associated with deficits in memory functioning, potentially through hypervigilance or attentional biases that interfere with encoding and retrieval [7]. The results in the literature are mixed, but some conclusions can be drawn. Impairments in memory performance are rather linked to a high level of state anxiety than to a high level of dispositional anxiety [13]. This implies that anxiety is much more likely to be the primary mechanism behind memory impairment than proneness to stress [7].

### 1.4. Memory Disruptions in Depression

A global memory impairment was noticed in patients diagnosed with depression compared with a group of healthy individuals [14]. This impairment seemed to affect both short‐term and long‐term memory [15]. It has been proved that episodic memory is negatively impacted in unipolar depression: people with depression report recollection deficits [16], enhanced memory performance for negative stimuli but impaired memory for positive elements [17, 18], and overgeneralization when retrieving autobiographical information [19, 20]. A relationship was found between the severity of depression and memory disruptions, which articulates that the more severe the symptoms, the greater the impairment is [15, 21, 22]. However, deficits in memory performance can subside during remission of depressive symptoms [23], whereas autobiographical retrieval can alleviate these symptoms [24]. This association points out that the relationship between memory performance and depression is bidirectional, and each influences the other [25].

### 1.5. Mnemonic Discrimination Performance: Pattern Separation

The essential functions of episodic memory include pattern separation and pattern completion associated with computations localized in the hippocampus. Memories can overlap, making it challenging to access them, so specific details are stored through pattern separation [26]. Researchers in the field suggest that pattern separation occurs during the encoding and possibly consolidation phases, primarily reducing overlaps between sensory inputs, thus making the resulting representations distinguishable [27]. Pattern separation is related to the specificity of memory [28], describing the extent to which a particular memory is specific, detailed, distinguishable, and unique. In contrast, pattern completion is the process of filling in partly overlapping, incomplete information using previously stored representations through the autoassociative network. These distinct computational procedures involve various subregions of the hippocampus. The dentate gyrus (DG) is associated with the process of pattern separation, while the cornu ammonis 3 (CA3) supports both pattern separation and completion. The specific engagement in either pattern separation or completion depends on its proximity to the DG and the dissimilarity of input [26]. The behavioral capacity known as mnemonic discrimination is derived from the process of pattern separation [29].

### 1.6. Theories and Behavioral Models

#### 1.6.1. Anxiety

The nature of the potential association between anxiety and mnemonic discrimination performance in humans is currently unknown [30]. To explain their relationship, multiple approaches have been proposed, including fear generalization. Fear generalization, by nature, is considered adaptive, as it allows us to adequately respond to new, potentially dangerous stimuli based on our previous experience with threat stimuli. However, individuals suffering from anxiety disorders tend to overgeneralize fear both in real life and in the laboratory [31], meaning they respond to safe elements or environmental cues the same way they do to dangerous ones. This phenomenon can be a phenotypic marker for anxiety disorders [32, 33]. Because pattern separation plays a role in stimulus discrimination, it was hypothesized that it is also responsible for discriminating safety stimuli that share common characteristics with threats [34]. If it functions properly, pattern separation can discriminate between safe and threatening stimuli. However, if this process is not successful, the representation of the threatening stimuli is activated by the safe ones, and this can lead to overgeneralization [34, 35].

Based on the abovementioned, we could assume that a general population would only have difficulties in hard mnemonic discrimination tasks, but those with anxiety would find easier tasks challenging as well [34].

#### 1.6.2. Depression

In animal experiments, mice with decreased neurogenesis showed impairment when stimuli were closely spaced but were unaffected when there was a greater spatial separation between them. This result suggests that newly generated neurons could be essential for pattern separation in the DG [36]. As neurogenesis is suppressed in depression, a deficit in pattern separation could be a potential explanation for poor recollection experienced by depressed individuals. Impaired pattern separation would prevent the person from successfully distinguishing representations during the encoding phase and overlapping representations during retrieval. In real life, a repeated retrieval of negative memories could sustain depressive episodes, and flawed encoding of positive memories can contribute to anhedonia. While this question is still open, the early results are promising [25].

### 1.7. Aims and Questions

Our systematic review is the first to study mnemonic discrimination and its associations to clinical and subclinical anxiety and depression. The aim of our review is to determine whether mnemonic discrimination, indicated by behavioral pattern separation performance, could be linked to the severity of anxiety and depression symptoms, and if so, in what way. Our review will summarize and interpret the empirical results to respond to the following questions: 1) Is mnemonic discrimination associated with anxiety and/or depression symptoms? 2) If so, how are they related? 3) Is there a difference in terms of nonclinical and clinical groups?

## 2. Method

### 2.1. Data Sources and Search Strategies

Before conducting the review, we registered our search protocols on PROSPERO (https://www.crd.york.ac.uk/prospero/display_record.php?ID=CRD42024502793). During the process, we followed the Preferred Reporting Items for Systematic Reviews and Meta‐Analyses (PRISMA) guidelines [37]. For the systematic search, we used fixed databases (PubMed, PsychINFO, Google Scholar, ScienceDirect, JSTOR, and SpringerLink) as well as artificial intelligence (Elicit). The search was conducted in February 2024, and studies published until December 31, 2023, were included. A manual search of reference lists in the selected articles was undertaken.

### 2.2. Inclusion and Exclusion Criteria

We aim to determine whether there are differences in mnemonic discrimination performance between individuals with lower and higher levels of anxiety and depression. Additionally, our goal is to elucidate the nature of the association between these variables, both in terms of direction and strength. Although our PROSPERO registration specified inclusion of studies using the Mnemonic Similarity Task (MST) [38], the literature search revealed a limited number of studies using the canonical three‐choice MST. Therefore, we broadened the inclusion to studies employing MST or closely related lure discrimination paradigms that assess behavioral pattern separation (e.g., emotional MST, CDR‐MST, BPS‐O, and DMS variants). This modification was made to capture the full scope of relevant evidence. Symptoms of anxiety and/or depression are assessed using any validated and recognized scales or inventories.

We only included quantitative studies. Our review encompasses both experimental interventions (between‐subject design) and observation studies that assess the natural variations in anxiety and depression levels among participants. We selected studies that clearly document the method of induction of anxiety or depression and measure its impact on or relationship with mnemonic discrimination performance. Those studies that measured anxiety or depression, but these constructs were not in the focus of the research, were excluded.

A transdiagnostic approach was applied when classifying anxiety disorders. Although these subdiagnoses show differences in anxiety response, they share common components and are comorbid [39]. Post‐traumatic stress disorder (PTSD) and obsessive‐compulsive disorder (OCD) were excluded for not being recognized by DSM‐V as anxiety disorders but obsessive‐compulsive and trauma‐related disorders [1].

An opposite approach was applied when searching for studies investigating depression. We only included studies with clinical samples that recruited patients with unipolar or major depressive disorder and excluded all other affective disorders.

An overview of the inclusion and exclusion criteria is provided in Table 1.

**Table 1:** Inclusion and exclusion criteria.

Inclusion criteria	1. Subclinical and clinical samples, 2. With adults, 3. With quantitative data, 4. Experience of diagnosed anxiety disorders/depression or elevated subclinical anxiety/depression symptoms (based on self‐report anxiety and depression scales), 5. Studies addressing pattern separation with elevated anxiety or depression symptoms, 6. Studies using validated self‐report measures of anxiety and depression, 7. Published in English, 8. In a peer‐reviewed journal or BSc/MSc/PhD thesis.
Exclusion criteria	1. Participants under 18, 2. If anxiety or depression was not in the focus of the study, 3. Studies inclusive of PTSD or OCD, 4. Studies where pattern separation was not studied directly, 5. Measuring anxiety in relation to a physical condition or illness (e.g., test anxiety), 6. Non‐peer reviewed books or reports.

As an example for exclusion, we excluded a study by Segal et al. [40] because they measured anxiety using salivary alpha‐amylase and did not use any validated self‐report measures for anxiety.

### 2.3. Screening, Data Abstraction, and Synthesis

The database search results were recorded and tracked in an Excel spreadsheet, from which duplicates were removed. One reviewer (AK) screened records for inclusion based on predefined criteria, and the other reviewer (SK) checked decisions. The reviewers did not identify any discrepancy in their judgments, but, in the event of a disagreement, an additional third‐party mediator and an external expert would have been involved to provide an impartial perspective and mediate the disagreement. The extracted data included author, year, location, study design, participant demographics, methodology, measures, analysis, and results. We then synthesized the data and explored emerging patterns.

### 2.4. Quality Assessment

The quantitative quality assessment was done at both the study and outcome levels. We undertook two types of assessment: levels of evidence and quantitative quality assessment. The National Health and Medical Research Council (NHMRC) Evidence Hierarchy [41] was applied to evaluate the rigor of research study designs. We used the NIH Study Quality Assessment of Systematic Reviews and Meta‐Analyses [42] to assess internal validity in studies selected for the review. Both reviewers were involved in the quality assessment.

## 3. Results

### 3.1. Anxiety

After the initial screening of databases (checking the aforementioned platforms), we identified 7475 studies, out of which duplicates were excluded. The screening process involved filtering articles based on titles, abstracts, and keywords, followed by a thorough examination of the full texts. As a result, nine studies met the criteria, which were included in the synthesis.

#### 3.1.1. Characteristics

##### 3.1.1.1. Location and Time

The majority of the studies (*n* = 8; 88.8%) were conducted and published in the USA, 8 of them (89%) were based on university or community samples. All the studies were born within the last 10 years, with 6 (66.6%) being published in the last 5 years. The characteristics and data of the studies are presented in Table S1 in the Appendix.

##### 3.1.1.2. Participants

In total, the nine studies included a participant pool of 864 individuals (339 males, 525 females). All participants were adults, ranging in age from 18 to 50 years. The average age across the studies was 29.23 years. Most of the studies (*n* = 7, 78%) did not report the ethnicity of the participants. Those that did included 59%–80% Caucasian, 4%–17% Black or African American, 13%–17% Asian or Asian American, 6%–12% Latino or Hispanic participants, and 4%–7% participants of other ethnicities. All participants were recruited from universities or community settings.

##### 3.1.1.3. Mental Health Status and Diagnoses

The populations examined by the studies were all community/nonclinical samples with one exception [43]. Anxiety levels were assessed using validated questionnaires, self‐report measures, structured clinical interviews (*n* = 1; 11%), and/or physiological indicators (*n* = 3; 33%).

#### 3.1.2. Levels of evidence

This systematic review included nine studies investigating the relationship between mnemonic discrimination and anxiety, comprising four Level II randomized controlled trials, one Level III‐1 pseudo‐randomized trial, one Level III‐2 comparative observational study with concurrent controls, and three Level IV cross‐sectional studies. According to the NHMRC Evidence Hierarchy, this body of literature provides moderate‐ to high‐quality evidence. Level of evidence ratings can be found in Table 2.

**Table 2:** Levels of evidence and quality ratings of anxiety studies.

Study	NHMRC Evidence Hierarchy	NIH quality assessment result
Balderston et al. [30]	Level II	Fair
Bernstein and McNally [44]	Level IV	Good
Bernstein et al. [45]	Level III‐1	Good
Caulfield et al. [46]	Level III‐2	Fair
Cunningham et al. [47]	Level II	Fair
Dohm‐Hansen and Johansson [48]	Level IV	Fair
Granger et al. [43]	Level IV	Good
Jiang et al. [49]	Level II	Fair
Ponzini and Steinman [50]	Level II	Good

*Note:* NIH, National Institute of Health Quality Assessment Tool for Observational Cohort and Cross‐Sectional Studies [42].

Abbreviation: NHMRC, National Health and Medical Research Council [41].

#### 3.1.3. Quantitative Quality Assessment

Based on the NIH criteria, the studies reviewed had fair to good study quality (see details in Table 2).

#### 3.1.4. Synthesis of Results

1. Is mnemonic discrimination associated with anxiety symptoms?

Of the nine studies, six found a significant association between memory discrimination and severity of anxiety symptoms. Only studies that used validated self‐report measures in addition to possible physiological measures were included in the review process.

##### 3.1.4.1. Studies With Stress Induction

Balderston et al. [30], Cunningham et al. [47], and Jiang et al. [49] obtained mutually corroborating, consistent results. These authors investigated the effect of acute stress on memory discrimination performance. In their experiments, stress was induced in members of the stress conditioning group using threat of shock [30] and the Trier Social Stress Test [47, 49]. Balderston et al. [30] and Jiang et al. [49] increased participants’ stress levels during the encoding phase. Both research groups concluded that memory discrimination performance is best when encoding is threatening and retrieval is in a safe environment. People who had both encoding and retrieval in threatening conditions did not perform significantly differently from the control group, despite their cortisol levels also being elevated.

Cunningham et al. [47] used stress induction after the encoding phase, with retrieval occurring the next day. They reported that, compared to the control group, members of the stress group were significantly more efficient at discriminating negative valence images. However, they also pointed out that the relationship was not linear but rather U‐shaped, based on the participants’ cortisol levels: moderate cortisol levels following stress were also associated with increased pattern discrimination for negative memory images.

In contrast, Bernstein et al. [45] found different results. Examined in a within‐subject design, with all subjects tested in both stress‐free (control) and stressful situations induced by psychological stressors, memory discrimination performance was worse after the application of social‐evaluative stressors, but this was not associated with anxiety symptom severity measured by DASS‐21. In Ponzini and Steinman’s [50] study, neither trait (social anxiety) nor state anxiety (stressor condition) predicted mnemonic discrimination performance.

##### 3.1.4.2. Studies With No Stress Induction

The studies that did not use stress induction report different and divergent results.

When the similarity between two memory traces is high, and thus memory discrimination is more difficult, individuals with higher levels of trait anxiety perform significantly worse [46]. Caulfield et al. [46] used the STAI measure of anxiety, but Dohm‐Hansen and Johansson [48] were only able to replicate their results on a larger sample size (70 participants) using the BAI measure; they found no significant relationship between nonclinical/subclinical anxiety symptom severity and mnemonic discrimination performance with the STAI. The difference in results may be due to several factors. Dohm‐Hansen and Johansson [48] used a paradigm for measuring memory discrimination that also considered the context of the objects, which may require more capacity and performance compared to the standard paradigm. In addition, Caulfield et al.’s sample had a lower mean score for anxiety symptoms as measured by the STAI. Their study also accounted for stimulus similarity, which was not a factor in Dohm‐Hansen and Johansson’s [46].

Other research has also found no relationship between mnemonic discrimination performance and anxiety severity using the DASS‐21 measure [44]. It is possible that the discrepancy in results may be because the DASS‐21, unlike the STAI, does not accurately measure behavioral inhibition, a significant construct in the maintenance of anxiety [46].

A study involving an emotional mnemonic discrimination task also found no relationship between anxiety (as measured by the BAI) and either neutral or negative stimuli [43]. While the factor analysis successfully distinguished healthy individuals from those with a diagnosis, it was unable to differentiate between depression and depression comorbid with anxiety. The heterogeneity of the clinical group may therefore limit the validity and generalizability of the study’s findings.

2. Is there a difference in terms of nonclinical and clinical groups?

While the studies reviewed here have examined various levels of anxiety, only one of them [43] compared a clinical group with a control group. This study found no relationship between anxiety and emotional behavioral pattern separation performance. However, it reported a heterogeneous clinical sample in which individuals diagnosed with depression could not be reliably distinguished from those with depression comorbid with anxiety. Since only one study included a comparison between a clinical and a control group using such a heterogeneous clinical sample, drawing definitive conclusions is challenging. Moving forward, it is crucial to examine different anxiety disorders separately rather than as a mixed group within clinical research.

### 3.2. Depression

After the initial screening of databases, we identified 10,847 studies, out of which duplicates were excluded. The screening process involved filtering articles based on titles, abstracts, and keywords, followed by a thorough examination of the full texts. As a result, 14 studies met the criteria, which were included in the synthesis.

#### 3.2.1. Characteristics

##### 3.2.1.1. Location and Time

The majority of the studies (*n* = 8; 57%) were conducted and published in the USA, 10 of them (71%) were based on university or community samples. Eleven studies (79%) were born within the last 10 years, with 5 (36%) being published in the last 5 years. The characteristics and data of the studies are presented in Table S2 in the Appendix.

##### 3.2.1.2. Participants

In total, the 14 studies included a participant pool of 1300 individuals (564 males, 736 females). All participants were adults, ranging in age from 18 to 90 years. The average age across the studies was 23.8 years. Most of the studies (*n* = 10, 71%) did not report the ethnicity of the participants. Those that did included 42%–80% Caucasian, 5%–17% Black or African American, 5%–22% Asian or Asian American, and 6%–33% Latino or Hispanic participants, among other ethnicities. All participants, including the ones with diagnoses, were recruited from universities or community settings.

##### 3.2.1.3. Mental Health Status and Diagnoses

The populations examined by the studies were all recruited from universities and communities, including the ones with a diagnosis. Depression levels were assessed using validated questionnaires, self‐report measures, and physiological indicators (*n* = 3; 21%). None of the studies employed structured clinical interviews in their assessments.

#### 3.2.2. Levels of Evidence

The body of literature identified in this review comprises 14 studies examining the relationship between mnemonic discrimination and depression. According to the NHMRC Evidence Hierarchy, this includes four comparative observational studies with concurrent controls (Level III‐2) and 10 cross‐sectional studies (Level IV). While no randomized controlled trials were identified, the available evidence provides valuable preliminary insight into this emerging area of research and establishes a foundation for future studies employing higher‐level designs. Level of evidence ratings can be found in Table 3.

**Table 3:** Levels of evidence and quality ratings of depression studies.

Study	NHMRC Evidence Hierarchy	NIH quality assessment result
Bernstein and McNally [44]	Level IV	Good
Camfield et al. [51]	Level III‐2	Good
Déry et al. [52]	Level IV	Fair
Dohm‐Hansen and Johansson [48]	Level IV	Fair
Fujii et al. [53]	Level IV	Fair
Granger et al. [43]	Level IV	Good
Grupe et al. [54]	Level IV	Good
Hayes et al. [55]	Level III‐2	Good
Komber and Tukiainen [56]	Level IV	Fair
Leal et al. [57]	Level III‐2	Fair
Leal et al. [57]	Level IV	Fair
Phillips et al. [58]	Level III‐2	Good
Semenova et al. [59]	Level IV	Fair
Shelton and Kirwan [60]	Level IV	Fair

*Note:* NIH, National Institute of Health Quality Assessment Tool for Observational Cohort and Cross‐Sectional Studies [42].

Abbreviation: NHMRC, National Health and Medical Research Council [41].

#### 3.2.3. Quantitative Quality Assessment

Based on the NIH criteria, the studies reviewed had fair to good study quality (see details in Table 3).

#### 3.2.4. Synthesis of Results

1. Is mnemonic discrimination associated with depression symptoms? If so, what is the direction and strength of the relationship?

Of the 14 studies, nine found a significant association between memory discrimination and severity of depression symptoms. Only studies that used validated self‐report measures in addition to possible physiological measures were included in the review process.

Although there are conflicting results in the literature, most studies point in one direction. Elevated levels of depression are associated with poorer mnemonic discrimination performance [52, 53, 59, 60], and the effect of depressive symptoms selectively affects pattern separation, with less generalized effects on hippocampus‐dependent memory [52]. FMRI studies also support that the activity of DG/CA3 and CA1 lateral hippocampal subfields involved in pattern separation during the similar item discrimination task is impaired by subclinical depression [53]. These findings are further supported by the fact that the association between more severe depressive symptoms and poorer discrimination performance is reduced as expected when taking an antidepressant [60]. The results of Grupe et al. [54] partially support this: individuals with low levels of depressive symptoms had better pattern separation performance than others, but they could not demonstrate this relationship with high levels of depressive symptoms. It is worth noting, however, that Grupe’s study only examined the anhedonic component of depression.

These results are supported and clarified by studies that used emotionally charged images instead of neutral stimuli. The mnemonic discrimination performance of participants with depressive symptoms is impaired for neutral stimuli but selectively enhanced for negative pictures. This negative bias is associated with depression severity [55, 57, 61], but the former can be reduced by distancing [55]. Consistent with this, clinically diagnosed individuals who responded well to antidepressant treatment show reduced negative bias and better pattern discrimination performance for neutral stimuli than those whose condition improved little or not at all with medication, but this difference is only present for images with low similarity [58].

Komber and Tukiainen [56], while not obtaining significant results, were able to show a trend in the same direction as above. They measured mnemonic discrimination performance using the Nordin’s colored squares test rather than pictures, as is usual in the literature. Camfield et al. [51] also found no relationship between depression status and pattern separation performance, but those with more severe depressive symptoms (including control group members) showed a reduction in the accuracy of discrimination of similar stimuli. Granger et al. [43], who also compared the performance of the diagnosed group with a control group, also found no results; in their case, the problem was that the factor analysis could not separate those diagnosed with depression from those diagnosed with depression and anxiety. Bernstein and McNally [44], who looked at the full continuum from mild to severe depressive symptoms, also failed to find a relationship.

2. Is there a difference in terms of nonclinical and clinical groups?

Only two studies have compared a clinical group with a healthy control group [43, 51]. Neither of these studies has shown a relationship between mnemonic discrimination performance and depressive symptom severity. The literature to date suggests that the relationship may be cross‐categorical, for example, Camfield et al. [51] found that the accuracy of pattern discrimination was reduced in those with more severe depressive symptoms, and this was also true for the control group. The question arises as to whether the diagnosed individuals were under medication, since, as demonstrated in the Phillips et al. [58] study, those who respond well to medication perform better on neutral stimuli than those who are nonresponders. It would certainly be worthwhile to design a study in the future that continues the research of Phillips et al. [58] by using a healthy control group to explore the differences between the groups.

3. Is there a difference across gender?

Among the studies reviewed, only one identified differences across gender. The analysis revealed that female participants outperformed men on the mnemonic discrimination task despite the fact that there was no significant difference in depression levels between the two genders [59]. The male–female ratio in the sample was 62% and 38%, respectively. In contrast, Leal et al. [57, 61] found no differences regarding any of the variables. While caution should be exercised in drawing conclusions from these findings, they underscore the importance of further investigation into this area, suggesting avenues for future research.

## 4. Discussion

### 4.1. Anxiety and Mnemonic Discrimination

The above leads to the conclusion that there is no consensus in the literature or clear results on our questions. This is presumably due to the lack of consistency in the methodology of the studies: (1) different measures are used, which may capture different aspects of anxiety (STAI [46–49], BAI [48], DASS‐21 [44, 45], and SUDS [50]); (2) different methods are used to induce stress (TSST [45, 47, 49], threat of shock [30], and potential public performance [50]); (3) the time of retrieval is different for the memory discrimination task: following the encoding phase [30, 44–46, 48, 50] or the next day [47, 49]; (4) most studies used neutral [30, 44–46, 49, 50], while others used emotionally charged pictures [47] or an object‐context paradigm [48].

Although we cannot draw a single conclusion, we can see a trend in the results. For stress induction studies, we see that the majority of studies (60%) found that memory discrimination performance is best when encoding happens in a threatening environment and retrieval happens in a safe environment. Two studies (40%) did not find significant results, which may be due to a lower mean level of anxiety in the sample or even a failure to induce sufficient levels or duration of anxiety compared to authors who obtained positive results [45].

Overall, the results also depended on the instrument used to measure anxiety. No studies using the DASS‐21 measure [44, 45] showed a relationship between anxiety severity and mnemonic discrimination performance, while studies using the STAI showed a relationship with one exception [48]. This phenomenon should be considered when designing further research, as it may suggest that the two measures capture different aspects of anxiety, which may play a different role in memory discrimination. Within our included corpus, we found no examinations of panic/agoraphobia, specific phobias, separation anxiety, intolerance of uncertainty, or rumination in relation to mnemonic discrimination.

To summarize the results, it is recommended that a unified methodology be pursued in this area, preferably involving clinical groups, to better understand the relationship between trait and state anxiety and mnemonic discrimination performance.

### 4.2. Depression and Mnemonic Discrimination

Most of the studies summarized in the review support each other; their results are consistent and point in the same direction. The methodologies used in the studies are much more homogeneous than in the case of anxiety. With few exceptions [44, 54, 56, 60], all studies used the original or the revised version of the BDI to assess the severity of depressive symptoms. Given these factors, it can be concluded with relative certainty that the literature suggests that depressive symptom severity is negatively related to pattern separation performance, that is, the more severe an individual’s symptoms, the harder and worse it is for them to separate similar stimuli [52, 53, 59, 60]. It is also found that pattern separation is enhanced for negative stimuli and impaired for neutral stimuli in people with depression [55, 57, 61].

However, there is no clear difference between clinically diagnosed groups and healthy controls [43, 51], which may be due to several factors: the effects of medication and possible heterogeneity of the clinical group. Of course, it is possible that there are other reasons for this, which we have not yet identified. In any case, it would be important to investigate this issue further to explore possible factors.

Further research is needed to explore gender differences. To the best of our knowledge, only one study identified differences across gender, which has shown that women perform better on mnemonic discrimination tasks even when there is no difference in the severity of depressive symptoms between men and women [59]. It is not possible to draw conclusions from a single study, but it is certainly worth investigating further.

### 4.3. Recognition Memory

Although pattern completion was not the primary focus of our review, we checked whether studies reported it given its complementarity to pattern separation. Across papers, a dedicated pattern completion index was rarely computed; most reported standard recognition (targets/foils) and sometimes used it as a covariate. Notably, Dohm‐Hansen and Johansson [48] derived an overgeneralization index, whereas Bernstein et al. [45], Granger et al. [43], and Shelton and Kirwan [60] reported recognition measures without a specific completion index. Overall, several studies reported recognitions, but few quantified pattern completion per se.

Across the four papers, recognition/pattern completion shows little consistent linkage to anxiety or depression. In Bernstein et al. [45], anxiety‐related effects on memory were specific to mnemonic discrimination and persisted after controlling for recognition, with no significant recognition‐anxiety association reported. In Dohm‐Hansen and Johansson [48], the mental health relationships centered on discrimination metrics, with no reliable associations for recognition highlighted. By contrast, Granger et al. [43] found that higher anxiety was linked to worse recognition of negative items, and this remained significant when age and sex were covariated. However, the correlation between depression and negative recognition did not survive covariate adjustment. Finally, Shelton and Kirwan [60] reported that higher depression predicted poorer pattern separation, while no parallel recognition deficit was emphasized in the abstract.

### 4.4. Theoretical Integration

The studies summarized in this review collectively indicate that both anxiety and depression are associated with alterations in mnemonic discrimination. Yet, the mechanisms and behavioral expressions of these changes appear to diverge. From a neurocognitive perspective, these differences can be conceptualized within a shared framework of hippocampal pattern separation dysfunction, modulated by stress and affective valence.

Mnemonic discrimination relies on computations in the DG and CA3 subfields of the hippocampus, which generate orthogonalized memory traces to reduce interference between similar experiences [26, 27]. Both chronic stress and depressive states are linked to structural and functional changes in these regions, including reduced neurogenesis and impaired DG–CA3 connectivity [25, 36]. Consequently, emotional or physiological stressors that dysregulate hippocampal activity can bias mnemonic computations toward pattern completion, whereby partial cues trigger the retrieval of overlapping negative representations rather than distinct new ones.

Across stress‐induction paradigms, anxious states appear to modulate mnemonic discrimination in a context‐dependent manner. When encoding occurs under threat and retrieval in safety, discrimination performance improves, possibly reflecting adaptive arousal‐mediated enhancement of DG efficiency [30, 49]. In contrast, sustained or trait anxiety—particularly when safety cues are ambiguous—reduces discrimination accuracy, consistent with fear generalization models [31, 32]. Deficient pattern separation may, therefore, contribute to difficulty distinguishing safe from threatening cues, maintaining hypervigilance and avoidance in daily life. This interpretation aligns with evidence that anxiety primarily affects state‐dependent encoding and high‐similarity lure discrimination, suggesting that the neural threshold for treating a cue as “novel” versus “threat‐related” is shifted toward generalization.

In depression, deficits in mnemonic discrimination are more consistent and exhibit a valence‐specific asymmetry. Individuals with higher depressive symptom severity show reduced discrimination for neutral stimuli but enhanced discrimination for negative stimuli [52, 55, 57, 61]. This pattern likely reflects an interaction between impaired hippocampal differentiation and amygdala‐driven bias toward negative material. Reduced DG neurogenesis limits the formation of distinct positive or neutral memories, while intact or hyperresponsive amygdalo‐hippocampal pathways selectively strengthen negative representations. The resulting negative memory bias reinforces rumination and pessimistic expectancy, thereby perpetuating depressive symptomatology. Preliminary evidence that antidepressant response normalizes mnemonic discrimination for neutral stimuli [58] further supports the mechanistic relevance of hippocampal plasticity in affective recovery.

Together, these findings support a transdiagnostic framework in which anxiety and depression represent distinct manifestations of a shared hippocampal‐limbic imbalance. Anxiety involves contextual overgeneralization of threat due to dynamic modulation of DG/CA3 processing under acute stress, whereas depression entails a chronic reduction in mnemonic specificity driven by neurogenic and valence‐dependent alterations in hippocampal encoding. Both pathways converge on impaired cognitive–emotional differentiation, leading to maladaptive patterns such as excessive avoidance, rumination, and reduced behavioral flexibility. Conceptually integrating these mechanisms highlights mnemonic discrimination not merely as a correlational marker but as a functional bridge between neural computation and clinical symptom expression. This perspective strengthens the theoretical coherence of the literature and underscores the potential of pattern‐separation paradigms as cognitive biomarkers and intervention targets in affective disorders.

### 4.5. Limitations

Although this review followed PRISMA guidelines and incorporated an extensive, multi‐database search strategy, the potential for publication bias and reporting bias cannot be excluded. Studies yielding null or contradictory findings regarding mnemonic discrimination in anxiety or depression may be underrepresented in the published literature, leading to an overestimation of effect consistency and magnitude. Recent meta‐analytic frameworks, such as that proposed by Lin and Chu [62], allow estimation of the proportion of unreported null results and demonstrate that even modest bias can substantially distort observed effect distributions. While the present review did not perform such quantitative modeling, awareness of these approaches underscores that our conclusions should be interpreted cautiously, as the true relationship between mnemonic discrimination and affective symptoms may be weaker or less consistent than implied by the published record.

Moreover, several included studies lacked full transparency in reporting methodological details, such as recruitment procedures, stimulus characteristics, or task parameters, raising the possibility of selective outcome reporting. Although we aimed to include only studies published in English due to language barriers, we did not identify any relevant studies that were published in another language.

In addition, there was considerable heterogeneity in methodological approaches across studies, including differences in the version of the MST (object vs. context vs. emotional variants), stimulus valence (neutral vs. negative), scoring metrics (e.g., LDI vs. d′), timing of retrieval (immediate vs. delayed), and psychometric instruments used to assess anxiety and depression (e.g., STAI, BDI, and DASS‐21). These discrepancies complicate direct comparisons and may account for inconsistencies across results. Furthermore, the predominance of university‐based and nonclinical samples and limited representation of clinical populations restrict the generalizability of findings to broader or more severe patient groups. A detailed overview of the psychometric instruments and their original sources is provided in the Appendix [63–75].

Future research should prioritize standardized experimental paradigms, preregistration of hypotheses, and open data sharing to mitigate bias and facilitate quantitative synthesis. Including clinical, diverse, and medication‐controlled samples will be critical for establishing whether mnemonic discrimination deficits represent a robust and generalizable cognitive marker of anxiety and depression.

### 4.6. General Importance of the Topic

Research on the association of mnemonic discrimination with anxiety and depression is considered a relatively new, unexplored, and understudied area. Yet, its investigation is significant for several reasons. Depression and anxiety are not only the two most widely experienced mental illnesses but also have a high comorbidity. Given that a significant proportion of the population may be affected by these problems, it would be important to understand whether high levels of depression and/or anxiety, even at subclinical or clinical levels, are associated with other changes in memory, particularly in relation to mnemonic discrimination. This would not only provide a more comprehensive understanding of the functioning of affected individuals but also answer the question of the role these conditions play in the alteration of perception and memory, specifically in the ability to discriminate between similar memory traces.

Deficits in mnemonic discrimination have tangible implications for daily functioning. Individuals who cannot effectively distinguish similar, yet safe contexts may exhibit persistent anxiety in benign situations, undermining social and occupational functioning. Likewise, reduced memory specificity in depression can impair problem‐solving, future planning, and emotion regulation, contributing to functional disability and relapse risk. Understanding these mechanisms may therefore inform clinical assessment and treatment development. Behavioral pattern‐separation tasks, such as the MST [38], could serve as cognitive biomarkers to identify individuals at risk, monitor treatment response (e.g., antidepressant efficacy, psychotherapeutic progress), and tailor interventions aimed at restoring memory specificity and reducing maladaptive generalization.

As evident from our summary above, the literature shows contradictions for both disorders, although, in the case of depression, we see relatively clear results pointing in one direction. It would be particularly important to clarify the situation with a unified framework and methodology, because utilizing the results in practice could complement and sensitize diagnostic processes through the application of behavioral pattern separation tasks. Similarly, individuals could receive broader, more satisfactory care by targeting cognitive changes.

## Author Contributions

**Alexandra Kaszás**: conceptualization, database search, data analysis, quality assessment, original draft. **Szabolcs Kéri**: conceptualization, validation, review, editing and corrections, supervision.

## Funding

The research conducted did not receive funding from any external sources.

## Conflicts of Interest

The authors declare no conflicts of interest.

## Supporting Information

Additional supporting information can be found online in the Supporting Information section.

## Supporting information


**Supporting Information** Table S1. Summary of methodological characteristics of studies on the relationship between anxiety and pattern separation included in the review, including study design, setting, participant characteristics, anxiety type and measures, stress induction procedures, and key findings. Table S2. Summary of methodological characteristics of studies on the relationship between depression and pattern separation included in the review, including study design, setting, participant characteristics, anxiety type and measures, stress induction procedures, and key findings.

## Data Availability

Data sharing is not applicable to this article, as no datasets were generated or analyzed during the current study.
