# Supplementary material for: Mnemonic Discrimination Performance in Anxiety and Depression: A Systematic Review
Source: Depress Anxiety. 2026 Feb 4;2026:8826935. doi: 10.1155/da/8826935 (PMC12869273; doi:10.1155/da/8826935)
Supplement: Supplementary file 1 — Supporting Information Table S1. Summary of methodological characteristics of studies on the relationship between anxiety and pattern separation included in the review, including study design, setting, participant characteristics, anxiety type and measures, stress induction procedures, and key findings. Table S2. Summary of methodological characteristics of studies on the relationship between depression and pattern separation included in the review, including study design, setting, participant characteristics, anxiety type and measures, stress induction procedures, and key findings. [file DA-2026-8826935-s001.docx]

**Appendix**

**Supplementary Table 1 - Characteristics of studies on anxiety included in the review**

| **Study and Settings** | **Participant Characteristics** | **Type of Anxiety and Measures** | **Stress Induction** | **Key findings** |
| --- | --- | --- | --- | --- |
| Balderston et al. (2015); USA | Non-clinical (community).  N=21; 17 female; age range: NA (M=29.4 yrs) | Affective scales | Threat of shock | - Greater anxiety observed during threat blocks compared to safe blocks, particularly during encoding (t(20) = 2.539; p = .02; dav = 0.314)  - Participants exhibited higher accuracy for old items compared to altered items during encoding than threat conditions (F(1, 20) = 25.401; p < .001; FDR = 0.005;h2 p = 0.559)  -Items retrieved during safe blocks showed better BPS compared to those retrieved during threat blocks (t(20) = −2.164; p = .043; FDR = 0.0774; dav = 0.619) |
| Cunningham et al. (2018); USA | Non-clinical (students).  N=50; 29 female; age range: NA (M=18.9 yrs) | STAI | TSST | - Participants exposed to the stressor exhibited increased cortisol concentrations compared to those in the control condition (*t*(48) = 3.8, *P* < 0.0001  - The stress group experienced a significant increase in cortisol levels (*t*(27) = 5.4, *P* < 0.001)  - Participants in the stress group showed a significant rise in reported anxiety levels from baseline to post-TSST (*t*(27) = 5.7, *P* < 0.001)  - The interaction between emotion and group (F(1,48) = 5.0, P = 0.03) indicated an amplified mnemonic discrimination of negative objects within the stress group in contrast to the control group (*t*(48) = −2.7, *P* = 0.01)  - No difference was found across groups for neutral object discrimination (*t*(48) = 0.14, *P* = 0.89).  - The stress group exhibited heightened negative discrimination compared to neutral discrimination (t(27) = 2.4, P = 0.02), while no such distinction was noted in the control group (*t*(21) = −0.87, *P* = 0.4). |
| Bernstein & McNally (2018); USA | Non-clinical (community and students).  N=111; 75 female; age: 18-55 yrs (M=31.36 yrs) | DASS-21, PSWQ, SAM | No stress induction | - BPS score did not predict anxiety levels (F(1,108)=.78, p=.38)  - The interaction between state affect and BPS score significantly predicted anxious symptoms (F(3,105) = 4.40, p = .006, adjusted R2 = .09, 95% CI [.005, .22], B = -.02)  - No significant relationship was found between anxiety symptoms and BPS performance among participants with low PSWQ scores (ps>.05) |
| Bernstein et al. (2019); USA | Non-clinical (community).  Experiment 1: N=65; 39 female; age: 18-50 yrs (M=32.65 yrs)  Experiment 2: N=128; 77 female; age: 18-40 yrs (M=23.38 yrs) | DASS-21, PSWQ, SAM | TSST | - Participants who met criteria for GAD (M = 63.93, SD = 7.43) reported higher scores (M = 44.39, SD = 13.67), t(125.79) = 10.47, p < .0001.  - Valence ratings remained consistently lower in the stress condition compared to the control condition at Time 2 (mean difference = –1.01, p < .01), Time 3 (mean difference = –0.69, p < .01), and Time 4 (mean difference = –0.99, p < .01).  - Main effect of condition: mean SCL was higher during the stressor visit compared to the control visit, F(1, 522) = 21.77, p < .001  - There was no group differences at baseline (mean difference = .003, p = 1.0) but significant group differences emerged during the manipulations (speaking mean difference = .24, p = .003; counting mean difference = .24, p = .004) and showed similar trends during the MST (encoding mean difference = .13, p = .14; retrieval mean difference = .15, p = .08).  - Significant differences were observed when age was controlled at 20 (p = .01) or 30 (p = .04), but no differences were found at ages 40 or 50 (ps > .05). When analyzing only participants under 40, a significant relationship between condition and LDI score emerged (F(1, 42) = 9.96, p = .003, b = −5.93, marginal R2 = .02, conditional R2 = .81)  - There was no main effect of LDI score (F(1, 54) = .05, p = .83, or condition by LDI interaction, F(1, 52), p = .11) |
| Jiang et al. (2019); USA | Non-clinical.  N=80; all male; age: 18-30 yrs (M=19.95 yrs) | STAI | TSST | - STAI scores did not exhibit differences among the control group, consolidation - stress group, and retrieval - stress group (F(2,66) = 0.72, P = 0.49)  - The analysis of consolidation - stress and control and response revealed a significant interaction between pre- and post-stress STAI scores and group (F(1,46) = 65.5, P < 0.001).  - An interaction between the retrieval - stress group and the control group in pre- and post-stress STAI score was also evident in the analysis of group and response. (F(1,43) = 29.92, P < 0.0001).  - 10 min after the TSST, STAI scores significantly increased in both the consolidation - stress group (t(23) = 5.73, P < 0.0001) and the retrieval - stress group (t(20) = 6.37, P < 0.0001) compared to the respective STAI scores before the TSST.  - The analysis revealed a significant interaction (F(1,40) = 16.33, P < 0.001) between pre- and post-stress cortisol levels and group  - The pre- and post-stress cortisol levels exhibited an interaction between the retrieval - stress group and the control group (F(1,39) = 23.38, P < 0.0001).  - Participants in the consolidation - stress condition demonstrated a significantly higher tendency to correctly label lure items as “similar” compared to participants in the control condition, who more frequently labeled lure items as “old” (t(46) = 4.88, P < 0.0001))  - The retrieval - stress group showed no significant difference in lure responses compared to the control group (t(43) = 1.08, P = 0.29)  - More lure items were identified as “similar” by the consolidation - stress group compared to the control group across all lure bins (1-5 bins) (t(46) = 3.98, P = <0.001; t(46) = 2.36, P = 0.02; t(46) = 2.21, P = 0.03; t(46) = 6.35, P < 0.001; t(46) = 2.90, P < 0.01) |
| Ponzini & Steinman (2020); USA | Non-clinical (students).  N=130; 82 female; age: 18-35 yrs (M=20.82 yrs) | Mini-SPIN, SUDS, Behavioral avoidance task | Future speech task | - A signiﬁcant interaction  between SUDS and condition was found (F(5, 625) = 7.03, p< .001) and  between SUDS and group (F(5, 625) = 10.01, p< .001)  - SUDS scores signiﬁ-  cantly diﬀered right after stress induction, meaning that the stress condition had higher SUDS scores than the control group (t  (128) = 3.82, p< .001)  – SUDS scores  diﬀered right after stressor re-  induction, indicating that the stress condition had higher SUDS scores than the control group (t  (128) = 2.62, p= .01)  - SUDS scores did not diﬀer after the encoding phase (p= .22) and after the retrieval  phase (p= .22)  - Individuals with  high social anxiety had signiﬁcantly higher  SUDS scores than individuals with low social anxiety  (p < .00) |
| Dohm-Hansen & Johansson (2020); Sweden | Non-clinical (students).  Experiment 1: N=30; 19 female; age: 19-31 yrs (M=22 yrs)  Experiment 2: N=87; 71 female; age: 18-38 yrs (M=22 yrs) | STAI, BAI | No stress induction | Study 1: - STAI-T, BDI and LDI: no significant correlations were found  Study 2:  - decrease in LDI scores correlates with an increase in overgeneralization (rs = .249; p = 0.021)  - As for BAI, no difference was found between object and context LDI similar (t(84) = -0.393, p > 0.05) |
| Caulfield et al. (2021); USA; | Non-clinical (students).  N=83; 58 female; age: 18-22 yrs (M=19.41 yrs) | STAI, AMBI, RMBI | No stress induction | - Hard similar items were harder to distinguish from previous items (t(59) = 13.230, p<.001)  Mnemonic discrimination:  - easy similar items, no significance: R2=.054, F(2,31)=.877, p=.426, AMBI ß=-.258, p =.227, STAI-trait ß =.227, p=.287)  - hard similar items, significance: R2=.220, F(2,31)=4.368, p=.021, AMBI ß=-.408, p =.040, STAI-trait ß =.548, p=.007)  Mnemonic efficiency:  - easy similar items, no significance: R2=.053, F(2,31)=.872, p=.428, AMBI ß=-.015, p =.944, STAI-trait ß =-.222, p=.297)  - easy similar items, no significance: R2=.245, F(2,31)=5.033, p=.013, AMBI ß=-.414, p =.035, STAI-trait ß =.-584, p=.004)  - LDI and AMBI: ß=.278, p=.162  - LDI and STAI-Y: ß=-.517, p=.012 |
| Granger et al. (2022); USA | Non-clinical (community) and clinical.  N=79 (22 healthy + 57 diagnosed); 58 female; age: 18-41 yrs | BAI | No stress induction | - Diagnosis and emotional valence for LDI do not correlate (F4,189 = 0.50, *P* = 0.74)  - Poorer LDI performance for positive stimuli compared to neutral ones (F2,216 = 5.40, *P* = 0.005)  - Overall anxiety symptoms showed no association with LDI for negative (rs = -0.19, *P* = 0.10) or neutral (rs = -0.10, *P* = 0.39) items |

*Note: STAI: Spielberger State-Trait Anxiety Inventory [63]. TSST: Trier Social Stress Test [64]. DASS: Depression Anxiety and Stress Scale [65]. PSWQ: Penn State Worry Questionnaire [66]. SAM: Self-Assessment Manikin [67]. Mini-SPIN: Mini Social Phobia Inventory [68]. SUDS: Subjective Units of Distress Scale [69]. BAI: Beck Anxiety Inventory [70]. AMBI/RMBI: Adult and Retrospective Measures of Behavioural Inhibition [71]. MASQ-30: 30-item Mood and Anxiety Symptoms Questionnaire [72].*

**Supplementary Table 2 - Characteristics of studies on depression included in the review**

| **Study and Settings** | **Participant Characteristics** | **Measures for depression** | **Key findings** |
| --- | --- | --- | --- |
| Déry et al. (2013); Canada | Non-clinical (students).  N=57; 39 female; age range: NA (M=19.48 yrs) | BDI | - People with lower levels of depression were better at correctly identifying lure items [t(50) = 2.23, p = 0.03]  - Individuals with lower levels of depression were better at distinguishing less similar objects from old items [t(50) = 2.34, p = 0.01]  - High levels of depression are associated with the decreased ability to correctly identify lure objects as similar within the same block as the original target [r(50) = -0.272, p = 0.05] and across blocks [r(50) = -0.297, p = 0.03] |
| Shelton & Kirwan (2013); USA | Non-clinical (students).  N=98; 67 female; age: 17-58 yrs (M=20.79 yrs) | DASS | - The members of the low depression group showed a better pattern separation performance compared to the high depression group [t(70) = 2.56, p = 0.013]  - Stimulus similarity had a main effect on the correct identification of lure stimuli [F(1,70) = 138.17, p < 0.001)  - The low depression group were better at distinguishing similar lures from old compared to the high depression group [t(70) = 2.87, p = 0.005] |
| Komber & Tukiainen (2013); Sweden | Non-clinical (students).  N=40; 26 female; age range: NA (M=24 yrs) | MADRS-S | - No relationship between pattern separation and depression |
| Fujii et al. (2014); Japan | Non-clinical (community).  N=23; 13 female; age range: NA (M=22.52 yrs) | BDI | - The more depression an individual experienced, the less active their DG/CA3 regions were during the encoding phase (P = -0.44, p < 0.05)  - In the same regions, the level of depression were negatively associated with the BOLD signal in all similarity conditions (low: P = -0.28, p = <0.10; middle: P = -0.40, p = 0.05; high: P = -0.0.38, p < 0.05)  - The BOLD signal and the severity of depression showed a negative correlation in the lateral CA1 region as well during the encoding phase (left lateral: P = -0.53, p < 0.01; right lateral: P = -0.54, p < 0.01) |
| Leal et al. (2014a); USA | Non-clinical (students) and clinical (students).  N=28 (18 healthy + 10 diagnosed); 16 female; age range: NA (M=21.22 yrs) | BDI-II | - Emotion and group showed a significant interaction [F(2,52) 5 3.28, P 5 0.046]  - Individuals in the depression group outperformed healthy individuals in discriminating emotional lures [F(1,52) 5 5.36, P < 0.05]  - Individuals with depression exhibited reduced activity for negative compared to neutral lure discrimination trials [F(1,26) 5 8.10, P < 0.05; critical Scheffe 5 4.23]  - Participants with depression showed higher activity for neutral compared to negative items, while the amygdala exhibited higher activity for negative compared with neutral items across groups [F(1,26) 5 12.27, P < 0.05].  - Depressive symptom severity was linearly associated with decreased activity in the amygdala [r 5 20.44, P 5 0.026] |
| Leal et al. (2014b); USA | Non-clinical.  N=130; 82 female; age: 18-35 yrs (M=20.82 yrs) | BDI-II | - LDI was reduced for emotional stimuli compared to neutral stimuli [F(1, 46) = 18.20, P < .001, critical Scheffé = 6.40]  - Low similarity lures were easier to discriminate than high similarity lures [F(1, 23) = 70.8, P < .001]  - LDI worsened after a 24-h delay compared to immediate testing [F(1, 36) = 46.78, P < .001]  - Differences in lure discrimination were greater for emotional stimuli compared to neutral stimuli over time [F(1, 72) = 6.28, P < .05, critical Scheffé = 6.24]  - Participants with depressive symptoms exhibited impairment in discrimination of neutral lures [F(1, 74) = 10.65, P < .05, critical Scheffé = 6.24], and enhancement in discrimination of negative lures [F(1, 74) = 12.55, P < .05, critical Scheffé = 6.24] |
| Semenova (2015); Sweden | Non-clinical (students).  N=82; 31 female; age: 19-37 yrs (M=22.9 yrs) | BDI-II | - BDI scores and pattern separation performance showed a weak negative correlation [r(80)= -0.14, p=0.036, 1-tailed, R2 = 0.019]  - Female participants outperformed male participants [ U= 571, z= -1.98, r=0.22, p=0.047, 2-tailed] in pattern separation |
| Berstein & McNally (2018); USA | Non-clinical (students and community).  N=111; 75 female; age: 18-50 yrs (M=31.36 yrs) | DASS-21 | - DASS Depression could not be predicted by the behavioral pattern separation score alone [F (1,108) = .15, p = .70]  - Poor behavioral pattern separation performance correlated with more severe depression at higher levels of negative affect, while stronger BPS performance correlated with mild symptoms to no depression [(B = −.08, p = .13)]  - Individuals with severe worry and more severe symptoms of depression had lower behavioral pattern separation scores [(B = −.10, p = .047)], while no relationship was found between the performance and depression among people with low levels of worry (ps > .05). |
| Camfield et al. (2018); Australia | Non-clinical (community) and clinical.  N=76 (44 healthy + 32 diagnosed).  Age group 1: N=52; 38 female; age: 18-35 yrs (M=21.23 yrs)  Age group 2: N=24; 9 female; age: 55-90 yrs (M=74.24 yrs) | BDI | - In the elderly group, the elevated level of depression was associated with poorer lure accuracy [r(19) = −0.519, p = .016], as well as decreased reaction time [r(19) = −0.499, p = .021] |
| Dohm-Hansen & Johansson (2020); Sweden | Non-clinical (students).  Experiment 1: N=30; 19 female; age: 19-31 yrs (M=22 yrs)  Experiment 2: N=87; 71 female; age: 18-38 yrs (M=22 yrs) | BDI | - LDI and BDI showed no significant correlations  - The LDI similarity of objects is negatively related to the level of depression [rs = -.235; p = 0.028]  - A positive association was found between overgeneralization for objects and BDI scores (rs = .278; p = 0.009)  - The object-in-context lure discrimination was not more sensitive to depression than only object lure discrimination (max t(84) = -0.235, p > 0.05) |
| Grupe et al. (2022); USA | Non-clinical (Mturk).  N=515; 229 female; age: 18-60+ yrs (M=39.2 yrs) | MASQ-30 | - Perceived stress implicated increased lure discrimination when the level of anhedonic depression was controlled [*t*(89) = −3.37, *p* = 0.001, *b* = −0.009, 95% CI [−0.014, −0.004]]  - The level of anhedonic depression is positively related to lure discrimination when perceived stress level is controlled for [*t*(89) = 4.05, *p* < 0.001, *b* = 0.010, 95% CI [0.005, 0.016]]  - For individuals with lower level of depression, the level of perceived stress is related to worse LDI scores [*r*(49) = −0.41, *p* = 0.003, 95% CI [−0.62, −0.15]] |
| Granger et al. (2022); USA; community and clinical | Non-clinical (community) and clinical.  N=79 (22 healthy + 57 diagnosed); 58 female; age: 18-41 yrs | BDI-II | - There was no significant interaction between diagnosis and emotional valence for LDI performance [F(4,189) = 0.50, *P* = 0.74]  - Participants performed worse in pattern separation for positive stimuli than for neutral stimuli [F(2,216) = 5.40, *P* = 0.005; *adjusted P* = 0.0034] |
| Hayes et al. (2023); USA | Non-clinical (students and community).  N=85; 60 female; age range: NA (M=20.20 yrs) | BDI-II | - The level of depression in the Control and the Distancing groups differed significantly [Distancing: t = -10.27, p <.001, Cohen’s d = -3.00; Control: t = -7.71, p <.001, Cohen’s d = -2.50]  - Greater depression severity was associated with higher negative affect ratings [r = 0.29, p =.01  - Negative affect ratings are positively related to the level of depression [r = 0.18, p =.09]  - In individuals who applied psychological distancing, negative lure discrimination was reduced and neutral lure discrimination was enhanced [F(1,22) = 4.48, p =.04, ηp2 = 0.17] |
| Phillips et al. (2023); USA | Clinical (responders vs. non-responders).  N=48; 34 female; age: 18-35 yrs (M=21.5 yrs) | BDI-II | - Performance in neutral lure discrimination was better than in emotional lure discrimination [*F*(1,46) = 45.79, *p* < 0.001, *ηp*2 = 0.50]  - Lures with low similarity were better discriminated than those with high similarity [*F*(1,92) = 199.58, *p* < 0.001, *ηp*2 = 0.81]  - For low similarity lures, it was easier to discriminate neutral lures than emotional lures [*F*(2,92) = 12.53, *p* < 0.001, *ηp*2 = 0.21], while for high similarity lures, it was more difficult to discriminate negative lures than positive or neutral ones [*F*(1,46) = 20.54, *p* < 0.001, *ηp*2 = 0.31] |

*Note: BDI: Beck Depression Inventory [73]. DASS: Depression Anxiety and Stress Scale [65]. MADRS-S: Montgomery-Åsberg Depression Rating Scale Self-Assessment version [74]. BDI-II: Beck Depression Inventory revised version [75]. MASQ-30: 30-item Mood and Anxiety Symptoms Questionnaire [72].*
